# The evolution of virulence of West Nile virus in a mosquito vector: implications for arbovirus adaptation and evolution

**DOI:** 10.1186/1471-2148-13-71

**Published:** 2013-03-20

**Authors:** Alexander T Ciota, Dylan J Ehrbar, Amy C Matacchiero, Greta A Van Slyke, Laura D Kramer

**Affiliations:** 1 Wadsworth Center, Arbovirus laboratory, New York State Department of Health, 5668 State Farm Road, Slingerlands, NY, 12159, USA; 2 Department of Biological Sciences, State University of New York, 1400 Washington Ave, Albany, NY, 12222, USA; 3 School of Public Health, State University of New York at Albany, One University Place Rensselaer, East Greenbush, NY, 12144, USA

## Abstract

**Background:**

Virulence is often coupled with replicative fitness of viruses in vertebrate systems, yet the relationship between virulence and fitness of arthropod-borne viruses (arboviruses) in invertebrates has not been evaluated. Although the interactions between vector-borne pathogens and their invertebrate hosts have been characterized as being largely benign, some costs of arbovirus exposure have been identified for mosquitoes. The extent to which these costs may be strain-specific and the subsequent consequences of these interactions on vector and virus evolution has not been adequately explored.

**Results:**

Using *West Nile virus* (WNV) and *Culex pipiens* mosquitoes, we tested the hypothesis that intrahost fitness is correlated with virulence in mosquitoes by evaluating life history traits following exposure to either non-infectious bloodmeals or bloodmeals containing wildtype (WNV WT) or the high fitness, mosquito-adapted strain, WNV MP20 derived from WNV WT. Our results demonstrate strain-specific effects on mosquito survival, fecundity, and blood feeding behavior. Specifically, both resistance to and infection with WNV MP20, but not WNV WT, decreased survival of *Cx. pipiens* and altered fecundity and bloodfeeding such that early egg output was enhanced at a later cost.

**Conclusions:**

As predicted by the trade-off hypothesis of virulence, costs of infection with WNV MP20 in terms of survival were directly correlated to viral load, yet resistance to infection with this virulent strain was equally costly. Taken together, these results demonstrate that WNV MP20 infection decreases the transmission potential of *Cx. pipiens* populations despite the increased intrahost fitness of this strain, indicating that a virulence-transmission trade-off in invertebrates could contribute significantly to the adaptive and evolutionary constraint of arboviruses.

## Background

Virulence, the fitness cost to a host resulting from pathogen infection, is a dynamic trait fluctuating with the co-evolution of both host and pathogen as well as with their interactions with changing environments. Although newly emergent pathogens are often more virulent, and many pathogens have displayed decreased virulence over time [1], the avirulence hypothesis, the idea that pathogens should always evolve away from virulent interactions with their hosts, has been largely disproven by epidemiological and experimental data demonstrating the persistence and/or evolution of highly virulent pathogen strains [2,3]. Despite this, although vector-borne pathogens are often associated with high virulence in vertebrate hosts [4], interactions between arthropod vectors and arthropod-borne viruses (arboviruses) have historically been characterized as benign [5-7]. Although the term *vector* implies a lack of significant biological interaction between arthropods and the pathogens they carry, it has become clear in recent years that such interactions are complex and are likely dominant forces shaping the evolution of arboviruses [8-11].

The alternative to the avirulence hypothesis is the trade-off hypothesis, which proposes that virulence and transmission are coupled and that the extent of virulence at equilibrium is subsequently limited by the trade-off that maximizes pathogen transmissibility [12]. Although variability in modes of transmission, intrahost competition, and relationships between virulence and pathogen load for individual host-pathogen systems may argue against the broad applicability of this hypothesis to explain variations in virulence [13], the trade-off hypothesis nevertheless provides a useful framework by which to evaluate the capacity for virulence evolution in individual systems. The coupling of virulence and transmission has indeed been noted in many systems [3,14,15], yet to-date has not been evaluated for an arbovirus in an invertebrate host.

Arboviruses, which are almost exclusively mosquito-borne RNA viruses with inherently vast evolutionary potential, have been relatively slow to evolve [16-19]. This evolutionary constraint has been attributed primarily to the obligate cycling between disparate vertebrate and invertebrate hosts [20-22], yet the effect of host cycling may at times be overstated [23-26] ; and genetic bottlenecks both within and among hosts and seasons [27-30], as well as cooperative interactions among variants [31] could also contribute to dampened rates of adaptation and evolutionary change in nature. In addition, a coupling of viral fitness and vector virulence could add further to this evolutionary constraint.

Although mosquito-borne viruses which rely heavily on vertical transmission for maintenance are generally not thought to be highly pathogenic to their invertebrate hosts, significant effects on life history traits of mosquitoes have at times been associated with infection of horizontally transmitted viruses generally associated with human disease [32]. Fitness costs in terms of decreased survival or fecundity, as well as tissue-specific pathology have been noted with both Alphaviruses [33-38] and Flaviviruses [39-41]. *West Nile virus* (WNV; *Flaviviridae*: *Flavivirus*,), the most geographically widespread arbovirus in the world, is vectored primarily by *Culex* mosquitoes and maintained in an enzootic cycle between these mosquitoes and avian hosts. Previous studies have demonstrated species-specific differences in the costs of WNV resistance and infection in *Culex* mosquitoes which correlate with variation in WNV vector competence [41]. Specifically, colonized *Culex tarsalis* exhibited fecundity costs associated with WNV infection but no cost for resistance [39], while *Cx. pipiens* demonstrated a cost for resistance but no cost associated with infection with wildtype WNV [41]. Experimental evolution studies with WNV previously generated a mosquito-adapted strain with both increased replicative ability and infectivity in *Cx. pipiens* [WNV MP20, [24]]*.* Here, by evaluating and contrasting life history traits of *Cx. pipiens* following exposure to either wildtype virus (WNV WT) or the mosquito-adapted WNV MP20 strain derived from WNV WT. We tested the hypothesis that virulence and viral fitness are coupled in vector-virus relationships, therefore limiting the capacity for arbovirus adaptation for higher levels of fitness in mosquito vectors. Our results provide a straight forward assessment of the relationships between intrahost viral fitness, virulence, and vectorial capacity which demonstrates that arbovirus adaptation and evolution could be profoundly influenced by strain-specific effects on life-history traits and transmission potential of mosquito vectors.

## Results

### WNV infection

**Figure 1:**
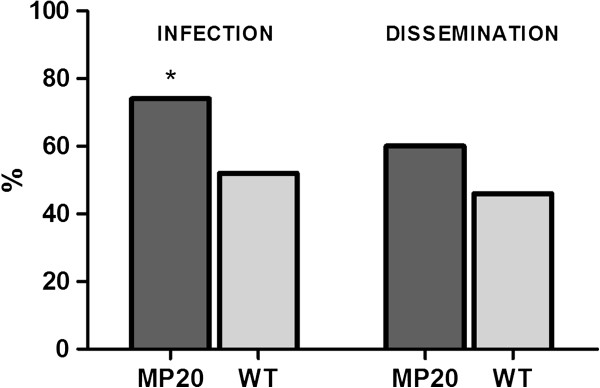
**Vector competence of *Cx. pipiens *following feeding on infectious bloodmeals containing either WNV WT or WNV MP20.** Infection refers to the percent of individuals with WNV positive bodies at the time of death and dissemination refers to the proportion of infected individuals with WNV positive legs at the time of death. *Fisher’s exact test, p<0.05.

**Figure 2:**
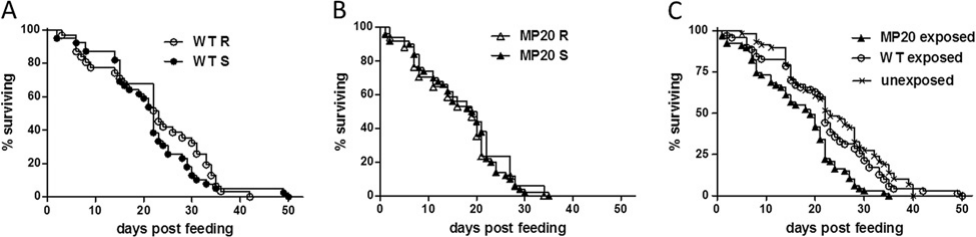
***Cx. pipiens *survival following blood feeding.** (**A**) Survival of WT-susceptible and WT-resistant *Cx. pipiens* following ingestion of WNV WT infectious bloodmeals. (**B**) Survival of MP20-susceptible and MP20-resistant *Cx. pipiens* following ingestion of WNV MP20 infectious bloodmeals. (**C**) Survival following bloodfeeding for WT-exposed, MP20-exposed, and unexposed *Cx. pipiens*. Significant differences in survival were identified between MP20-exposed mosquitoes and both WT-exposed and unexposed groups (log-rank, p<0.05).

**Figure 3:**
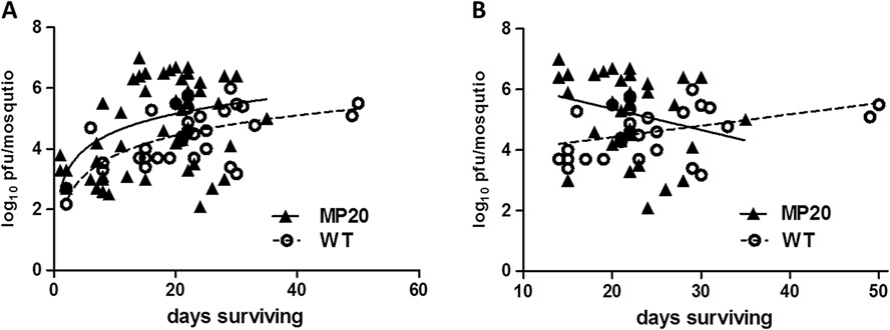
**WNV loads in *Cx. pipiens *at the time of death.** (**A**) Individual WNV titers for all infected mosquitoes and best-fit nonlinear relationship between survival and geometric viral titers. (**B**) Relationship between WNV titers and survival beginning at 14 days post-infection and best fit linear relationship between survival and geometric viral titers. Slopes of lines differed significantly (linear regression analysis, p=0.009) and a negative correlation between days surviving and viral load was measured for MP20-susceptible mosquitoes (Correlation analysis, Pearson r= −0.36, p=0.038).

Viral titers of infectious bloodmeals were 7.7 and 7.9 log_10_ pfu WNV/ml for WNV MP20 and WNV WT, respectively. Initial feeding rates were similar among groups, averaging ~55.0%. There were a total of 66, 67, and 70 fully engorged *Cx. pipiens* in the uninfected, MP20 and WT groups, respectively. WNV infection rates were significantly higher for mosquitoes exposed to MP20 relative to those exposed to WT (74.6% vs 55.7%; Fisher’s exact test, p=0.031; Figure 1). WNV loads at the time of death were 4.7 and 4.4 log_10_ pfu/mosquito for MP20 and WT groups, respectively, and log values were normally distributed (Shapiro-Wilk test and D’Agostino &Pearson test, p>0.1). Although this approximately 2-fold difference does not equate to statistically significant higher overall viral loads for the MP20 group when comparing geometric means (t-test, p=0.27), this difference is significant when comparing arithmetic means (t-test, p=0.015). Additionally, the variation in time tested (time of death) between both groups and individuals does not permit an accurate comparison of overall differences in viral loads. Specifically, earlier mean mortality of the MP20-infected mosquitoes (Figure 2) equates to fewer days of WNV replication in these individuals and an understatement of differences in viral load. Differences in replicative ability between WNV WT and MP20 are demonstrated by the fact that statistically different nonlinear curves were generated when comparing days surviving and viral loads, with a higher mean slope for the MP20 group (4.0), relative to WT (3.4; F test, F=4.39, p=0.039; Figure 3A). In addition, a significantly greater variation in body titers independent of time was measured for the MP20 group (F test, F=2.38, p=0.009; Figure 3). This variation decreased the power of any one model to fully explain these data, yet semilog curves provided a sufficient model to summarize the relationship between survival and viral load (replicates test, F=2.0, p=0.11 [MP20], F=1.8, p=0.08 [WT]). Dissemination rate, i.e., the proportion of infected individuals with WNV positive legs, was higher in the MP20-susceptible group, although the difference was not statistically significant (Fisher’s exact test, p=0.21; Figure 1) and again is confounded by variability in times of death. Significantly higher viral loads at the time of death were measured in MP20-susceptible mosquitoes relative to the WT-susceptible group when comparing individuals with disseminated infections (5.8 v. 4.9 log_10_pfu/mosquito; t-test, p=0.004), whereas the opposite relationship (higher viral loads in the WT group) was observed when comparing viral loads of individuals with non-disseminated infections (3.9 v. 4.9 log_10_pfu/mosquito; t-test, p=0.006).

A total of 5182 larvae, 2772 from the MP20-susceptible group and 2410 from the WT-susceptible group, were pooled and screened for WNV. A total of 7 pools were identified as WNV positive, 5 from WT-susceptible mosquitoes and 2 from MP20-susceptible mosquitoes. This equated to vertical transmission rates of 2.08 and 0.72 per 1000 larvae for WT and MP20-infected mosquitoes, respectively (Table 1). The 2 MP20 positive pools came from a single egg raft, whereas the 5 WT positive pools came from 4 egg rafts derived from 3 individual mosquitoes. All egg rafts from which positive larvae were derived were from the 3^rd^ or 4^th^ oviposition of individual females. In fact, all 5 egg rafts producing WNV positive larvae represented the only egg rafts from 3^rd^ or 4^th^ oviposits, demonstrating that WNV infected *Cx. pipiens* with the capacity to produce at least 3 egg rafts vertically transmitted the virus 100% of the time.

**Table 1:** **Vertical transmission of *West Nile virus *in *Cx. pipiens *following infection with WNV MP20 or WNV WT**

	**WNV MP20**	**WNV WT**
total larvae tested^1^	2772	2410
WNV+ pools	2	5
infection rate/1000^2^	0.72	2.08
WNV + rafts/total (%)	1/51 (1.96)	4/47 (8.51)
adults VT/ ovipositing (%)^3^	1/39 (2.56)	3/30 (10.0)
WNV+ OV 3–4 rafts/total^4^	1/1	4/4

^1^larvae were processed and tested in pools of 20–25.

^2^WNV+ pools were assumed to have a single positive individual.

^3^Refers to the proportion of ovipositing females vertically transmitting.

^4^Refers to the proportion of 3^rd^ or 4^th^ oviposition rafts which were WNV+.

### Mosquito fitness and virulence

In order to assess if alterations to *Cx. pipiens* fitness are associated with exposure to *Cx. pipiens*-adapted WNV, survival, fecundity, and wing size were assessed for individual mosquitoes exposure to MP20 and compared to both WT-exposed and unexposed groups.

Survival of both WNV-resistant and WNV-susceptible mosquitoes was similar within groups (log-rank, p=0.94 for MP20 [Figure 2A] p=0.39 for WT [Figure 2B]), permitting further comparisons based on exposure rather than infection status (Figure 2C). Decreased survival was measured in the MP20-exposed group relative to both the WT-exposed and unexposed groups (log-rank, p<0.001), whereas no difference in survival was associated with WT exposure relative to the unexposed group (log-rank, p=0.40; Figure 2C). Differences in survival are also significant when MP20-resistant and susceptible groups are compared individually to other groups (log-rank, p<0.001), demonstrating that a survival cost is associated with both establishment of, and resistance to, MP20 infection. Mean survival was 16.9 days post feeding (dpf) for the MP20-exposed group, relative to 21.9 and 23.8 dpf for the WT-exposed and unexposed groups, respectively. Maximum survival, which was 35 dpf for the MP20-exposed group, was also lower than both the WNV WT-exposed group (50 dpf) and the unexposed group (41 dpf). The survival cost resulting from exposure to WNV MP20 has been confirmed with subsequent experimentation (data not shown).

Although a WNV strain-specific difference in mosquito survival is clearly demonstrated here, the relationship between viral load and virulence (survival post exposure) was more difficult to assess with viral replication as a confounding factor when comparing days surviving and levels of WNV in mosquito bodies at the time of death (Figure 3). Despite this, when viral loads are compared to days surviving following the plateau of viral replication [~ 14 days, [24]] a negative correlation between days surviving and viral load is measured for MP20-susceptible but not WT-susceptible mosquitoes (correlation analysis, Pearson r= −0.36, p=0.038). Despite this relationship beyond 14 dpf, many MP20-exposed mosquitoes died prior to 14 dpf with relatively low viral loads or a complete lack of detectable infections (MP20-resistant group).

**Figure 4:**
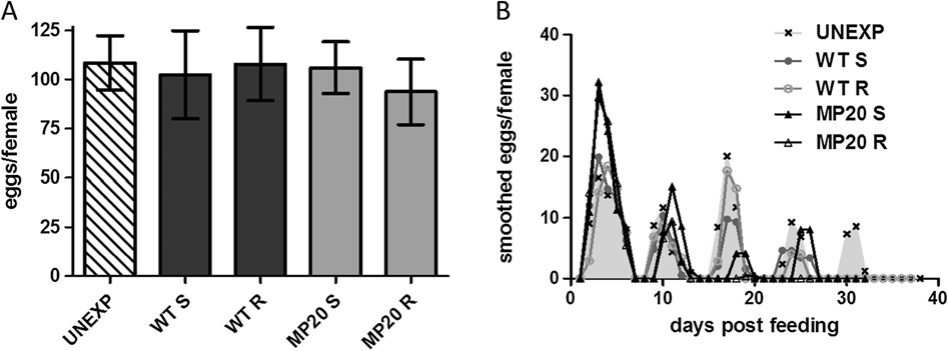
**Fecundity of *Cx. pipiens *following bloodfeeding.** (**A**) Mean eggs/female (**B**) The shaded region depicts fecundity of the unexposed mosquitoes (UNEXP).

Differences in fecundity among groups were assessed by comparing eggs/female, hatch rates, and larvae/female following exposure. Overall egg production did not differ significantly among groups (t-test, p>0.05; Figure 4A), yet patterns of reproductive output were highly variable depending on exposure and/or infection status (Figure 4B). Specifically, egg production/female was significantly higher in the MP20-exposed groups relative to WT-exposed or unexposed mosquitoes in the first week of the study (Figure 4B; t-test, p<0.01) and similar among groups in the 2^nd^ week. Following the 2^nd^ week of the study, a significant decline in eggs/female was measured for both WT-susceptible mosquitoes and, to larger extent, MP20-exposed groups, such that MP-20 exposed mosquitoes produced significantly fewer eggs relative to both WT-exposed and unexposed groups in weeks 3–6 (Figure 4B; t-test, p<0.01).

**Figure 5:**
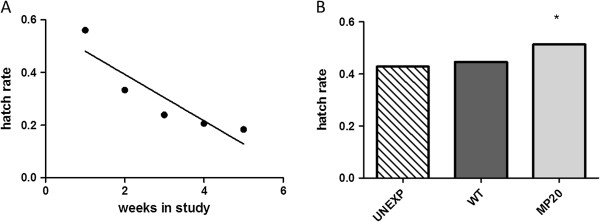
***Cx. pipiens *egg hatch rates.** (**A**)Combined weekly hatch rates for all groups. (**B**) Total hatch rates for individual groups. * Chi-squared, p<0.05.

As has been noted in previous studies [39], hatch rates declined with time (Figure 5A; linear regression analysis, r^2^=0.817, p=0.035). Overall, significantly higher hatch rates were measured in the MP20-exposed groups relative to WT-exposed and unexposed groups (Figure 5B; chi-squared, p<0.0001) and these differences are also significant when both MP20-susceptible and MP20-resistant groups are considered separately (chi-squared, p<0.0001). Since no significant differences in hatch rates among groups were measured for individual weeks (chi-squared, p>0.05), the increased hatch rate for MP20-exposed mosquitoes can be attributed wholly to differences in the timing of oviposition (i.e. more egg rafts produced early when hatch rates are high).

Larvae per female and probability of daily survival were used to produce life history tables to calculate net reproductive output (R_o_), generation time (T), and intrinsic rate of population increase (r) for experimental groups (Table 2). Although increased early reproductive output and increased hatch rates resulted in modestly higher mean larvae/female in the MP20-susceptible group, no significant differences were identified among groups (t-test, p>0.05). Values for R_0_ and r were also similar among groups, with the highest net reproductive output calculated for the unexposed group (52.8) and the lowest values for both R_0_ (46.3) and r (0.23) measured in the WT-resistant group. Generation times were similar for unexposed and WT exposed mosquitoes yet, consistent with the observation of increased early egg production, substantially lower for MP20-exposed mosquitoes (Table 2; Figure 4). There were no differences in mean wing length among groups, indicating that differences in life history traits and/or WNV susceptibility could not be attributed to mosquito size (Table 2).

**Table 2:** **Summary of mosquito fitness of*West Nile virus*susceptible (S), resistant (R), or unexposed*Cx. pipiens*following feeding on WNV WT, WNV MP20, or non-infectious (UNEXP) bloodmeals**

	**UNEXP**	**WNV WT S**	**WNV WT R**	**WNV MP20 S**	**WNV MP20 R**
N	66	39	31	50	17
wing (mm)^1^	3.57	3.59	3.56	3.57	3.58
MST (d)^2^	23.8	21.5	22.5	16.9	16.9
larv/female^3^	46.7+/−10.3	49.9+/−14.7	42.7+/−14.4	57.6+/−10.0	42.8+/−14.7
R_o_^4^	52.8	49.0	46.3	48.5	51.8
T (d)^5^	16.4	15.3	16.6	13.7	12.2
r^6^	0.31	0.26	0.23	0.28	0.32

^1^ mean wing lengths.

^2^ mean survival time.

^3^ mean larvae/female +/− SEM.

^4^ net reproductive output.

^5^ generation time.

^6^ intrinsic rate of population increase.

### Blood feeding

In order to assess if WNV exposure, infection, and/or virus strain altered blood feeding behavior in *Cx. pipiens*, weekly feeding rates and levels of engorgement were compared among groups. No differences in mean weekly feeding rates were measured , yet WNV infection significantly enhanced early (week 1) feeding rates relative to unexposed mosquitoes, particularly for MP20-infected mosquitoes (chi-squared, p=0.014; Table 3). Since there were more individuals early in the study, week 1 differences account for the modestly elevated overall feeding rates of infected individuals, yet rates of feeding beyond week 1 of the study increased for unexposed mosquitoes and decreased for WNV-exposed mosquitoes (Table 3). These differences in late feeding were significant when comparing individual MP20-resistant mosquitoes or combined MP20-exposed mosquitoes to unexposed mosquitoes (chi-squared, p<0.01). The proportion of females that imbibed at least one bloodmeal following the initial feeding to enter the study was significantly higher in the WT-exposed groups relative to all other groups (chi-squared, p<0.01; Table 3). This can be attributed to both slightly higher feeding rates in the WT-exposed groups relative to unexposed mosquitoes as well as significantly better survival relative to the MP20-exposed groups. Although not significant, this same trend, i.e. increased likelihood of feeding with WT-exposed mosquitoes, is evident when comparing the mean number of bloodmeals taken by individual mosquitoes in each group (Table 3). Levels of engorgement were also statistically equivalent among groups, yet volumes of blood ingested by fed mosquitoes were on average lower for the MP20-susceptible group, a result which may be biologically significant (Table 3).

**Table 3:** **Blood feeding behavior of *West Nile virus *susceptible (S), resistant (R), or unexposed (UNEXP) *Cx. pipiens *following feeding on WNV WT, WNV MP20, or non-infectious bloodmeal**

	**UNEXP**	**WT S**	**WT R**	**MP20 S**	**MP20 R**
overall rate^1^	0.29	0.39	0.29	0.37	0.24
wk. 1 rate^2^	0.20	0.46^a^	0.32	0.63^a^	0.43
wks. 2–6 rate^3^	0.41	0.31	0.28	0.23	0.10^b^
mean wk. rate^4^	0.23	0.32	0.27	0.28	0.15
w/o bm^5^	0.58	0.41^c^	0.42^c^	0.61	0.58
bm/female^6^	0.66	0.87	0.77	0.59	0.41
engorge^7^	2.61	2.27	2.46	2.08	2.29

^1^ proportion feeding throughout the study (total fed/total offered).

^2^ proportion feeding at 7 days post infection.

^3^ combined proportion feeding in weeks 2–6 of the study.

^4^ mean weekly feeding rates.

^5^ proportion of females not taking a bloodmeal throughout the study.

^6^ mean number of bloodmeals imbibed by individual mosquitoes.

^7^ mean level of engorgement (1–4).

^a^ significantly higher than unexposed (Chi-squared, p<0.05).

^b^ significantly lower than unexposed (Chi-squared, p<0.05).

^c^ significantly lower than all other groups (Chi-squared, p<0.05).

### Vectorial capacity

Vectorial Capacity (VC), i.e. the WNV transmission potential of this population of *Cx. pipiens*, was calculated for the WNV-exposed groups, using experimentally determined parameters, in order to assess if strain-specific differences existed. Since infection and dissemination, but not transmission were evaluated in this study, the product of these two values alone was used as a measure of vector competence (b, Table 4). Although not all mosquitoes with disseminated infections transmit virus, previous characterization of these strains failed to identify significant differences in WNV transmission once disseminated [24]. Additionally, using VC alone to compare the potential for population level expansion of individual strains (R_0_) assumes equivalent avian host susceptibility and levels of viremia, which have also been demonstrated in previous work evaluating infection and replication of these strains in chicks [24]. Results of the current study confirmed increased intrahost fitness of MP20 relative to WT, both in terms of replicative ability (Figure 3) and vector competence (b, Table 4; Figure 1). Regression analyses of survival curves of WNV susceptible mosquitoes demonstrates generally linear mortality with daily probabilities of survival (p) of 0.97 (r^2^=0.97) for MP20-susceptible mosquitoes and 0.98 for WT-susceptible mosquitoes (r^2^=0.88). Mean weekly blood feeding rates were used for the host feeding rate variable (h). Mean extrinsic incubation period (N) was estimated at 10 days based on previous studies [24,42,43]. Since the effect of population size was not evaluated here, mosquitoes/host (m) was not included in calculations of VC, resulting in a value representing the average transmission potential for each individual mosquito/host. Taken together, calculation of VC demonstrates that, despite adaptation for increased replicative ability and individual transmission potential, the population level transmission potential of *Cx. pipiens* exposed to the mosquito-adapted MP20 strain is lower than that of the WT-exposed population (Table 4).

**Table 4:** **Vectorial capacity (VC) of experimental populations of *Cx. pipiens *following exposure to WNV WT and WNV MP20**

	**h**_**1**_	**b**_**2**_	**p**_**3**_	**N**_**4**_	**VC= h**^**2**^**p**^**N**^**b/-ln(p)**
WNV WT	0.32	0.32	0.98	10	1.00
WNV MP20	0.28	0.44	0.97	10	0.83

_1_ mean blood feeding rate.

_2_ vector competence (b) = infection rate *dissemination rate.

_3_ probability of daily survival.

_4_ extrinsic incubation period (N) = mean time in days from infection to transmission.

## Discussion

As is the case with many pathogen-host systems, the capacity for transmission of arboviruses generally increases with increases in pathogen load in mosquitoes. For this reason, one would predict that, in the absence of opposing selective forces, evolution would favor maximal replicative fitness of arboviruses in mosquito vectors. Conventional wisdom predicts interaction of vectors and the pathogens they carry should generally be benign [44], implying that there should be little constraint on intrahost fitness in invertebrate hosts. Experimental evolution studies with *St. Louis encephalitis virus* (*Flaviviridae*: *Flavivirus*) demonstrate an inability for further adaptation to *Cx. pipiens,* suggesting this virus may indeed have achieved its evolutionary potential in this system [25], yet similar studies with WNV have demonstrated the capacity for further adaptation of this virus to *Cx. pipiens*[24]*.* Although there have been modest adaptive and consensus-level genetic changes since its introduction to the U.S., WNV, like many arboviruses, has remained remarkably static over time [42,45-48]. As has been shown with some systems, this could be partially attributed to differential selective pressures resulting from host cycling [16,21,22,49], yet studies with mosquito-adapted WNV MP20 demonstrate that host-specific adaptations without significant fitness trade-offs in vertebrate hosts are attainable, suggesting other adaptive constraints may exist [24]. In this study, using WNV MP20 and its parental strain (WNV WT), we show that arbovirus adaptation could be further constrained by the coupling of intrahost fitness and virulence in mosquitoes. Although a correlation between virulence and viral load, as well as strain-specific differences in vertebrate virulence for WNV and other arboviruses is well established [50-52], virulence of arboviruses in invertebrate systems has not previously been considered a dynamic trait contributing significantly to pathogen evolution. The lack of studies in this field is likely a result of the historic assumption that the invertebrate immune response is relatively generic, yet recent advances in mosquito genetics and invertebrate immunity have revealed complex interactions between vector-borne pathogens and their invertebrate hosts [10,53]. In addition, a number of studies have documented highly variable levels of arbovirus vector competence among populations of individual mosquito species, demonstrating the specificity of arbovirus-mosquito interactions and host-virus genotype by genotype outcomes [54,55]. This work establishes that strain-specific interactions with invertebrate hosts have the potential to be substantial forces shaping both vector and arbovirus evolution and adaptation.

Our results clearly demonstrate virulence resulting in decreased survival for MP20-exposed *Cx. pipiens* relative to both unexposed and WT-exposed *Cx. pipiens.* The fact that this decreased survival was not measured with WT-exposed mosquitoes demonstrates strain-specificity and establishes that virulence was a by-product of experimental evolution studies selecting for this high fitness strain [24]. For exposed individuals with detectable infections, a clear cost of infection was measured. In addition, a direct correlation between viral load and virulence beyond 14 days exposure was measured, demonstrating that arbovirus intrahost replicative fitness may be coupled with virulence in an invertebrate host. As predicted with the higher fitness *Cx. pipiens*-adapted MP20 strain, overall viral loads, as well as infection and dissemination rates were higher. Yet, interestingly, decreased survival for MP20-exposed mosquitoes was also measured for individuals with both relatively low levels of infection as well as with the MP20-resistant group, for which there were no detectable WNV infections. Previous studies with both WNV [41] and *Dengue virus*[40] have also demonstrated that fitness costs can be associated with resistance to infection , yet the cost for resistance measured in our study was measured only with MP20-exposed mosquitoes, demonstrating that the decreased fitness of resistant mosquitoes is not likely due simply to a coupling of WNV resistance and low fitness of mosquitoes, but instead a direct result of strain-specific exposure and, subsequently, defence against establishment of infection. These results suggest that the magnitude of invertebrate defence against establishment of arbovirus infection may be specific and that the cost for such defence may be directly correlated to strain virulence. Further studies investigating strain-specific immune response will be required to understand variation in the mechanisms and/or extent of immune gene activation that correlates with the costs of immune deployment. With RNA interference (RNAi) as the primary immune response to arbovirus infection in mosquitoes [56], it is possible that WNV MP20 elicits a more robust RNAi response which may be costly to the mosquito, yet this warrants future investigation. Regardless of mechanism, this result implies that chronic exposure of mosquito populations to arboviruses could have measurable effects on mosquito fitness and, subsequently, selective pressures, which are independent of vector competence. Recent studies in *Ae. aegypti* mosquitoes demonstrate that genes involved in RNA virus defence are subject to high levels of positive selection, suggesting a host-virus evolutionary arms race similar to what has been previously described for highly pathogenic microbes in vertebrate systems [57]

Previous studies with WNV have demonstrated a fecundity cost of infection for *Cx. tarsalis,* but not *Cx. pipiens* mosquitoes [41]. Although in the current study we again did not measure differences in overall fecundity in *Cx. pipiens*, our results do demonstrate an association between WNV infection and substantial alterations to reproductive patterns, particularly with MP20-exposed individuals. Specifically, MP20-exposed mosquitoes maximized early egg output at a later cost. This alteration in egg production could be viewed as an adaptation to maximize reproductive output in the face of decreased fitness, particularly since both bloodmeal digestion and egg production would be extremely costly for mosquitoes whose fitness is already compromised [58]. Indeed, increased early egg production resulted in increased hatch rates and similar overall reproductive output for WNV MP20-exposed individuals, despite both decreased survival and egg production beyond week 2 of the study. What is not considered in a controlled laboratory rearing setting is the uncertainty of successful feeding, oviposition and egg hatching, which in nature is largely dependent on fluctuating environmental conditions and likely maximized by producing multiple egg rafts over time [59]. For this reason, the reproductive strategy of MP20-exposed mosquitoes could be less productive in a natural setting. Another consequence of these altered reproductive patterns, at least from the perspective of the pathogen, is decreased probability of vertical transmission. Since *Culex* mosquitoes do not generally take a bloodmeal prior to overwintering [60] and vertebrate hosts are not known to be capable of developing significant persistent or recrudescent infective viremia [61,62], the capacity for vertical transmission of WNV in mosquitoes is likely critical for maintenance in temperate regions that experience significant seasonal breaks in transmission [63-65]. Since the majority of egg rafts derived from MP20-infected mosquitoes were produced following the first week of infection, the number of females vertically transmitting was lower than that of WT-infected mosquitoes. Although our overall rates of vertical transmission were low for both groups, and generally comparable to what has been previously reported [64,66-68], what was remarkable is that all 4 WNV-infected mosquitoes that produced a 3^rd^ or 4^th^ egg raft vertically transmitted. Since previous studies evaluating vertical transmission have generally been done *en masse*, without knowledge of the reproductive history of individuals producing positive larvae, it is possible that the potential for vertical transmission of WNV and other flaviviruses in mosquitoes have been significantly underestimated.

As with fecundity, overall blood feeding rates among groups were similar, yet differences in timing of bloodmeal acquisition, which could be significant in WNV transmission, were associated with both exposure and infection status. WNV infection significantly increased the likelihood of bloodmeal acquisition in the first feeding following exposure, particularly with the WNV MP20-exposed mosquitoes. As these mosquitoes are anautogenous, bloodmeal acquisition is a requirement for egg maturation; thus these differences generally correlate to fecundity differences and, like fecundity, early feeding enhancement results in a subsequent decrease in rates in the following weeks. Enhanced blood feeding with infection, as has been shown with WNV in *Cx. tarsalis*[39] as well as with Plasmodium infection of *An. gambiae*[69], could be viewed as a manipulation by the pathogen to increase transmission potential, yet since the likelihood of transmission increases with time, the decreased feeding beyond 7 days measured here, particularly with the MP20-exposed mosquitoes, is likely to instead decrease WNV transmission potential. This decreased probability of transmission could be further enhanced by the fact that, on average, MP20-infected mosquitoes consumed smaller bloodmeals, likely as a result of decreased feeding times.

The method of selection utilized in creation of the MP20 strain required only that a low proportion of surviving individuals transmit WNV to be used for subsequent passages [24]. These selection criteria, although sufficient to select strains with superior replicative fitness and, therefore, transmissibility, could also tolerate modest levels of virulence. Here, by calculating vectorial capacity, we have shown that this level of virulence would inhibit transmission potential on the population level, such that wildtype WNV would have an advantage in terms of invasion and population spread, despite the increased intrahost fitness of WNV MP20 in *Cx. pipiens*. This cost of virulence could contribute to the overall dampened rates of evolution and partially explain why similar ‘adaptive’ arbovirus strains do not emerge and persist as readily as would be predicted for pathogens with the capacity to so rapidly explore sequence space. The notion that individual strain fitness and vector virulence may be coupled, as predicted by the trade-off hypothesis and demonstrated here, could fundamentally change our understanding of how vector-virus interactions work to shape the evolutionary trajectories of arboviruses and other vector-borne pathogens.

What remains unknown is how variation in natural populations of mosquitoes may affect susceptibility to virulence. It is well documented that colonization can be detrimental to population fitness and it is possible that field populations could tolerate more fit strains without significant costs [70,71]. There are also likely to be significant temporal and generational variations in fitness which may have profound effects on vector-virus interactions and subsequent outcomes of infections. Differences in terms of the cost of resistance and blood feeding behaviour are evident when contrasting results presented here to previous evaluation of the effect of wildtype WNV exposure on *Cx. pipiens*[41]. Although these experiments were separated by ~4 years and it not surprising that significant changes to the colony population could occur over that time, this demonstrates that spatial and temporal variation precludes our capacity to make broad assumptions about the outcomes of vector-virus interactions. In whole, these results demonstrate that a greater comprehension of the complexity and specificity of interactions between vectors and pathogens will be required if we are to better characterize the evolution of these systems.

## Conclusions

The interactions between vector-borne pathogens and their invertebrate hosts have been characterized as being largely benign, yet the relationship between virulence and fitness of arthropod-borne viruses (arboviruses) in invertebrates has not been previously evaluated. Our results demonstrate, as predicted by the trade-off hypothesis of virulence, decreased survival of *Cx. pipiens* with increasing viral load of mosquito-adapted *West Nile virus* (WNV MP20), but not its parental strain, WNV WT, in terms of survival, as well as an equivalent cost for resisting infection with this strain. In addition, we have identified strain-specific influence on both bloodfeeding and reproductive patterns, with greater alterations resulting from exposure to the adapted strain. Taken together, these results demonstrate that infection with WNV MP20 significantly alters life-history traits and decreases the transmission potential of *Cx. pipiens* populations despite the increased intrahost fitness of this strain, indicating that a virulence-transmission trade-off in invertebrates could contribute significantly to the adaptive and evolutionary constraint of arboviruses. In whole, these results demonstrate that a greater comprehension of the complexity and specificity of interactions between vectors and pathogens will be required if we are to better characterize the evolution of these systems.

## Methods

### Virus strains and testing

Wildtype WNV (WT) was derived from WNV NY003356, a primary isolate from an American crow that was collected in 2000 in Staten Island [72] by plaque purification and amplification on Vero cells (ATCC #CCL-81) as previously described [23]. Mosquito-passaged and adapted WNV (MP20) was derived by passage of WNV WT 20 times in *Cx. pipiens* using intrathoracic inoculation and subsequent collection of salivary secretions for each passage as previously described [24]. Mosquito bodies, legs, and larval pools were collected in 1 ml mosquito diluent (20% heat-inactivated fetal bovine serum (FBS) in Dulbecco’s phosphate-buffered saline (PBS) plus 50 μg/ml penicillin/streptomycin, 50 μg/ml gentamicin, and 2.5 μg/ml Fungizone) and subsequently subject to homogenization and centrifugation as previously described [25]. All WNV screens and titrations for virus quantification were completed by plaque assay on Vero cell culture [73]. WNV-exposed mosquitoes refer to those fully-engorged with WNV containing bloodmeals, regardless of infection status. Resistant and susceptible mosquitoes were defined following virus exposure as those with or without established WNV infections at the time of death, respectively; given these experimental conditions, and therefore do not necessarily imply competence of mosquitoes when exposed to other WNV strains and/or doses.

### Mosquitoes

*Cx. pipiens* egg rafts were originally collected in Pennsylvania in 2004 (courtesy of M. Hutchinson) and colonized at the Arbovirus laboratory, Wadsworth Center. Mosquitoes were reared and maintained in 30.5 cm^3^ cages in an environmental chamber at 27°C, 50-65% relative humidity with a photoperiod of 16:8 (light:dark) hours. 300 adult mosquitoes (100 male/200 female) were collected for each exposure group upon emergence, held in mesh top 3.8 L paper cartons, and provided cotton pads with 10% sucrose *ad libitum*. Mosquitoes were held for 5–7 days prior to blood feeding to allow for mating.

### Blood feeding

Mosquitoes were deprived of sucrose for 24 hrs prior to blood feeding. Following starvation, females were distributed into three 0.6 L cups for experimental infections in the BSL-3 insectary. Mosquitoes were fed on defibrinated chicken blood (Rockland) with 2.5% sucrose together with either 20% BA-1 (unexposed; Hanks M-199 salts, 0.05 Tris pH 7.6, 1% bovine serum albumin, 0.035 g/l sodium bicarbonate, 100 units/ml penicillin, 100 mg/ml streptomycin, 1 mg/ml fungizone), WNV WT, or WNV MP20 . Virus strains were diluted to 8.5 log10 pfu/ml in BA-1 prior to bloodmeal mixing. Feeding was carried out for one hr using Hemotek membrane feeders (Discovery Workshops) heated to 37°C. Mosquitoes were then anesthetized using CO_2_ and fully-engorged females were separated and housed individually in cups containing oviposition dishes with 15mls of distilled water and access to 10% sucrose.

Subsequent uninfected blood meals were offered to all groups once a week for the duration of the study. Specifically, mosquitoes were again starved for 24 hrs and then offered pledgets soaked with chicken blood with 2.5% sucrose for one hour. Mosquitoes were monitored during these feedings and both numbers fed and degree of engorgement (1, small amount of blood in abdomen, no abdominal distention; 2, some distention, no pleural membrane observed; 3, significant abdominal blood, pleural membrane observed; 4, fully engorged, distended abdomen) were recorded.

### Mosquito fitness

Mortality and egg production were monitored and recorded daily for all groups. Wings were removed from dead mosquitoes, individually mounted on slides with double-sided tape, and measured as previously described using a Zeiss microscope, Axiocam camera, and Axiovision software [Carl Zeiss; [39]]. Individual mosquito bodies and legs were stored separately at -80^0^C and subsequently processed and tested for WNV as described above. Egg rafts were photographed under 50× magnification using a digital camera (Nikon) and digital images were used to count individual eggs.

Oviposition cups containing egg rafts were removed and held for approximately 2 days at 27°C to allow for hatching. Hatched larvae were provided with food (ground koi food: ground rabbit pellets, 1:1) and allowed to develop to 1^st^– 2^nd^ instar to permit counting and subsequent calculation of hatch rates. Larvae from individual rafts were combined in pools of 20–25, stored in MD at -80^0^C, and processed and tested as described for mosquito bodies.

### Data analysis

GraphPad Prism software version 4.0 was used for generation and analyses of survival curves. Statistical comparisons of curves were completed using a log-rank test. Both survival and reproductive data were used to construct life history tables for each group in separate replicates. Survival (l_x_) is equivalent to the proportion of mosquitoes surviving to day x, and reproductive output (m_x_) is equivalent to the number of eggs produced on day x. Data for m_x_ was smoothed by averaging individual daily egg output with the egg output on both previous and subsequent days. Reproductive output (total eggs produced in an average female’s lifetime; R_0_= ∑ l_x_ m_x_), generation time (average age at which females produce eggs; T=∑ l_x_ m_x_x/ R_0_), and intrinsic rate of increase (instantaneous population growth rate; r = ln R_0_/T) [74,75] were subsequently calculated. GraphPad Prism 4.0 was used for Chi-squared tests, Fisher’s exact tests, F tests, and correlation analyses, and Microsoft Excel was used to perform t-tests. Vectorial capacity (VC) is defined as the number of new hosts exposed to a pathogen by a specified population of mosquitoes per infected host per day [76,77]. VC= mh^2^p^N^b/-ln(p), where m = the number of mosquitoes/host, h = host feeding rate, p = the probability of daily survival, N = the mean extrinsic incubation period, and b = vector competence (proportion of exposed mosquitoes with disseminated infections).

## Competing interests

The authors declare that they have no competing interests.

## Acknowledgements

The authors would like to thank all of the members of the Arbovirus laboratory insectary staff for assistance with these studies and, particularly, Pamela Chin for mosquito rearing. This work was funded by the National Institutes of Health (NIH), grant number R01-AI-077669. The construction of the Wadsworth Center Insectary Facility was partially funded by NIH grant number C06-RR-17715.

## Authors’ contributions

ATC conceived and coordinated the experiments, analyzed and interpreted the data, carried out the experiments, and wrote the manuscript. DJE, GAVS, and ACM analyzed data carried out the experiments. LDK conceived and coordinated the experiments. All authors have read and approved the final manuscript.
